# A Monte Carlo based dose verification tool for an HDR Ir‐192 brachytherapy source and its evaluation on extremity malignancies

**DOI:** 10.1002/acm2.70778

**Published:** 2026-08-28

**Authors:** Peixiong Li, Chi Wah Kong, Rihui Zhang, Dejun Zhou, Chenyu Huang, Tin Lok Chiu, Pak Hang Nam, Chi To Yung, Tien Yee Chang, Siu Ki Yu, Bin Yang

**Affiliations:** ^1^ Department of Medical Physics Hong Kong Sanatorium & Hospital Hong Kong Hong Kong; ^2^ Medical Physics Graduate Program Duke Kunshan University Kunshan Jiangsu P.R. China

**Keywords:** dosimetry, extremity malignancies, interstitial brachytherapy, lower limb, monte carlo, sarcoma

## Abstract

**Background:**

TG‐43 formalism dose calculation for brachytherapy with the homogeneous water‐based assumption is widely used, which can lead to dose inaccuracy in treatment planning.

**Purpose:**

Extremity malignancies are also treated with high‐dose‐rate (HDR) ^192^Ir interstitial brachytherapy, a treatment site that has not been specifically investigated in prior comparative dosimetric studies, and the proximity of source dwell positions to bone and air introduces significant heterogeneity. BrachyPlanCheck, an in‐house brachytherapy second dose verification program for Monte Carlo (MC) simulations, incorporates these heterogeneities and is used for a comparative dosimetric study on extremity malignancies.

**Methods:**

BrachyPlanCheck was commissioned and validated in both a homogeneous water phantom and a heterogeneous breast phantom. The clinical evaluation included 8 patient‐specific lower limb brachytherapy plans, comparing TG‐43 and MC calculation using BrachyPlanCheck. Dosimetric differences were evaluated using dose comparisons, gamma index analysis, and dose‐volume histogram (DVH) metrics for clinical target volume (CTV) and organ‐at‐risk (OAR), including bone and skin.

**Results:**

In commissioning Level 1, MC simulations using BrachyPlanCheck demonstrated point dose agreement within ± 0.9%. Gamma passing rates exceeded 99.9% with criteria 1 mm/1% in commissioning Level 2. In clinical evaluation, TG‐43 calculation overestimated CTV V150 by up to 10.50% and CTV D90 by up to 7.25%. OAR doses were generally higher with TG‐43, with mean differences of 1.09% for bone D1cc (*p* =  0.109), 1.38% for bone D0.1cc (*p* =  0.078), 7.30% for skin D2cc (*p* =  0.008), and 5.68% for skin D0.1cc (*p* =  0.008).

**Conclusion:**

Validated by phantom and patient study, BrachyPlanCheck demonstrated robust performance for independent secondary dose verification. TG‐43 calculation overestimated dose to CTV and OAR in extremity malignancy cases compared with BrachyPlanCheck.

## 1 INTRODUCTION

Elekta treatment planning system (TPS) Oncentra (Elekta AB, Stockholm, Sweden) utilizes the AAPM TG‐43 formalism for dose calculation, which models radiation deposition using precomputed source data in homogenous water.^1^ Despite its computational efficiency and long‐standing clinical acceptance, the TG‐43 model neglects medium heterogeneity, which limits its use. Numerous comparative studies across a range of anatomic sites—including prostate, breast, skin, head and neck, esophagus, lung, liver, and gynecologic regions—have reported dose deviations between TG‐43 and more advanced algorithms such as model‐based dose calculation algorithms (MBDCAs) including ACE and Acuros BV, or MC simulations, often with TG‐43 calculation overestimating dose to both targets and OARs.^2^ Although AAPM TG‐186 reported minimal dose differences up to 5% within target volumes and up to 15% in OARs observed across breast, esophageal, and endorectal cases,^3^ larger discrepancies were reported in a nasopharyngeal cancer brachytherapy case. Specifically, TPS Oncentra utilizing TG‐43 formalism was found to overestimate the CTV D90 value by 40% when compared to MC simulation performed with *EGSnrc*.^4^ In addition, a comprehensive study focusing on cervical cancer cases reinforced this pattern across 7 different applicator techniques, with TG‐43 overestimating rectal D0.1 cm^3^ by up to 32%, D1.0 cm^3^ by 29%, and D2.0 cm^3^ by 28% compared with *EGSnrc*.^5^

These results warrant further investigation of dosimetry and comparison between different dose calculation methods. Therefore, an in‐house program called BrachyPlanCheck was developed with MATLAB (MathWorks, Natick, MA, R2022b) to perform secondary dose verification with the open‐source MC package *egs_brachy*, which is a flexible and efficient MC simulation tool for brachytherapy, built upon the *EGSnrc* code system.^6^, ^7^ Although MC simulation is time‐consuming compared with the TG‐43 method, it includes the real physical processes involved in the interaction of photons with matter in general and human tissues in particular, which is considered a gold standard in radiotherapy dose calculation. Compared with other tools such as ALGEBRA, BrachyGUI, and BrachyGuide, based upon Geant4, PTRAN, and MCNP, respectively, our program benefits from the optimized efficiency of egs_brachy.^8^, ^9^, ^10^

To evaluate this tool, although it applies to all intracavity and interstitial brachytherapy treatment sites, a dosimetric comparison between TG‐43 and BrachyPlanCheck is particularly performed on extremity malignancies treated with HDR interstitial brachytherapy cases, a treatment site that has not been specifically investigated in prior comparative studies, and the proximity of source dwell positions to heterogeneous tissues such as bone and air introduces significant discrepancy from the homogeneous water‐based assumptions of TG‐43. Furthermore, the presence of metal radiomarkers in CT‐based treatment planning, which are absent during treatment delivery, introduces additional uncertainty when heterogeneity is taken into account, as their dosimetric impact has not been systematically studied and may be clinically relevant.

## 2 MATERIALS AND METHODS

### 2.1 Patient characteristics

In our hospital, HDR interstitial brachytherapy using ^192^Ir source with TPS Oncentra is used to treat extremity malignancies, such as Kaposi sarcoma, and Myeloid sarcoma due to its highly conformed dose distribution and steep falloff around the target with better protection of subcutaneous tissues near the tumor, compared to external beam radiation therapy (EBRT). For Kaposi sarcoma, HDR brachytherapy is considered superior to EBRT in terms of dosimetric coverage, resulting in lower complication rates and offering a non‐surgical treatment option.^11^ BrachyPlanCheck evaluation was conducted using 8 patient‐specific lower limb brachytherapy plans with the institutional review approval of our hospital. The implant technique and treatment planning methodology have been previously described.^12^ All patients underwent HDR brachytherapy using ^192^Ir Flexisources (Nucletron, Elekta AB, Stockholm, Sweden) delivered via the Flexitron afterloading system (Isodose Control, Veenendaal, The Netherlands). Treatment plans were generated using the Oncentra TPS. The prescribed dose ranged from 42 to 55 Gy, administered twice daily over approximately 10 fractions. A median of 8 flexible plastic catheters ranging from 5 to 12 were implanted per patient, with a median of 174 dwell positions ranging from 69 to 280. Oncentra dose calculations for all plans were performed using the TG‐43 formalism, applying an isotropic dose grid resolution of 1 mm across the entire X‐ray CT imaging volume.

### 2.2 Material composition

In this study, patients’ CT images were processed using the *ctcreate* package to generate voxelized phantoms in *egsphant* format, compatible with the *egs_brachy*. For each simulation, two phantoms were constructed: one representing the full CT volume, and one centered around the source dwell region to define the dose calculation region. The center of the dose scoring volume was the centroid of dwell positions, which ensured focused dose evaluation while reducing unnecessary dose scoring. Voxel material assignment and mass density derivation are based on its HU value in the DICOM CT image. The assignment material list with corresponding nominal mass density range and maximum CT number in Hounsfield Units (HUs) used by *ctcreate* as shown in Table 1.^13^

**TABLE 1:** Materials assigned by BrachyPlanCheck.

Material Name	Max CT Number (HUs)	Density Range (g/cm^3^)
AIR_0.0012	−861	0.001–0.1306
LUNGS_0.26	−386	0.1306–0.605
ADIPOSE2_WW86	−12	0.605–0.985
MUSCLE2_WW86	110	0.985–1.075
CARTILAGE_WW86	670	1.075–1.475
CORTICAL_BONE_WW86	1500	1.475–2.2275

During CT simulation for treatment planning, metallic radiomarkers were visible to indicate catheter position, but were absent at treatment delivery. To investigate their impact on dose calculation, two steps of CT number override were implemented (MC_override_): (1)Water‐equivalent override: Voxels with CT numbers greater than 1500 HU located outside the contoured bone and skin within the body were grouped into clusters and classified as radiomarkers, which avoided overriding the catheter fixers and bone. For each cluster, the centroid was determined and expanded to a 3 mm diameter sphere to account for the blooming effect. All voxels within these spheres were reassigned to 0 HU, corresponding to water‐equivalent CT number, thereby eliminating dose perturbation from the high‐HU voxels while avoiding artificial air volume. (2)Air‐equivalent override: As a compromise, the catheter's inner lumen was contoured and directly overridden to −1000 HU (air‐equivalent). This adjustment compensated for the portion that should have been modeled as air but was otherwise overridden as water. If no override was applied, the calculation was performed using the phantom created from the original CT dataset (MC_original_).

### 2.3 BrachyPlanCheck

Although *egs_brachy* has its graphical user interface (eb_gui) developed in C++ using the Qt5 framework,^14^ to provide extra accessible alternatives, we developed BrachyPlanCheck with MATLAB to automatically generate input files for the open‐source *egs_brachy* MC package. The application was compiled and run independently on machines equipped with the MATLAB Compiler Runtime. BrachyPlanCheck extracted geometric and dosimetric parameters directly from the RT plan exported by TPS Oncentra. Source dwell positions were explicitly retrieved, and individual dwell times were derived based on the total dwell time and relative weighting factors. Key treatment parameters—including kerma strength (Gy·cm^2^/h), total dwell time, and maximum dwell weight—were involved in a dose scaling factor to obtain absolute dose in cGy, using the standardized air kerma strength per history of 1.16267 × 10^−^^1^ ^3^ Gy·cm^2^/history.^7^ Source orientation was interpolated from neighboring dwell positions along each catheter path to ensure accurate directional modeling. BrachyPlanCheck automatically applied *ctcreate* to convert the full CT dataset into two voxel‐based phantoms: one representing the complete patient anatomy and another localized subvolume centered at the geometric centroid of all dwell positions. This targeted calculation region neglected the relatively low dose zone to improve calculation efficiency without compromising accuracy. Dose was reported as $D_{m,m}$ where radiation transport or scatter‐integration calculations and dose‐specification were both performed with the phantom composition of the underlying voxels with the tracklength scoring method if not specifically assigned as recommended for high energy source by AAPM TG‐186.^3^ Output files generated by BrachyPlanCheck were formatted for compatibility with *egs_brachy*, enabling batch runs to make the most use of the processor and random‐access memory (RAM), simplifying phantom construction and calculation setup. For postprocessing, BrachyPlanCheck included a module to convert 3Ddose from *egs_brachy* into DICOM format, allowing integration with clinical platforms for visualization, benchmarking, and dosimetric analysis. Table 2 outlined BrachyPlanCheck MC features and input settings, in accordance with the guidelines provided by AAPM TG‐268.^15^ This end‐to‐end automation enabled high‐fidelity patient‐specific MC dose verification.

**TABLE 2:** Summary of the relevant MC characteristics and input parameters used in this study.

Category	Description	References
Code	*egs_brachy* (v2017.09.15) using egs++ from *EGSnrc* 2019 master branch	^7^
Validation	Validated in multiple studies	^16^, ^17^
Timing	∼3 h per patient‐specific dose calculation with a cluster of 28 cores split in 2 nodes of Intel(R) Xeon(R) CPU models: two E5‐2680 v4 @2.40 GHz	
Source Description	^1^ ^9^ ^2^Ir Flexisource	^18^
Cross‐Sections	XCOM; mass‐energy absorption data (precalculated)	^18^
Transport Parameters	Photons transported to 1 keV; no electron transport for tracklength scoring	^19^
Variance Reduction/ Efficiency	Absorbed dose approximated by collision kerma calculated using a track length estimator	^7^
Scored Quantity	Medium kerma in medium	
Histories/ Statistical Uncertainty	1 × 10^9^ histories (estimated with a reduced‐history pre‐run); up to 0.7% within CTV, 1% within bone, 2% within skin	^7^
Air‐kerma strength per history	1.16267 × 10^−^^1^ ^3^ Gy·cm^2^/history	^20^
Statistical Methods	History‐by‐history scoring	^7^
Postprocessing	Results interpolated to 1 mm^3^ isotropic resolution for direct comparison; egs_brachy output written to *.RD format compatible with MIM (MIM Software Inc., Cleveland, OH, USA, version 7.3.7)	

### 2.4 Validation method

#### 2.4.1 Commissioning level 1

The commissioning of BrachyPlanCheck followed a two‐tier protocol adapted from the AAPM Working Group on Dose Calculation Algorithms in Brachytherapy (WGDCAB)^21^. Level 1 validation involved absolute dose calculations for a single ^1^ ^9^ ^2^Ir Flexisource centered in a homogeneous water phantom (15×15×15 cm^3^) with dose scoring throughout the volume of 5×5×5 cm^3^ and 0.5 × 0.5 × 0.5 mm^3^ voxel size. MC calculations were performed using tracklength (TL). Dose values were then extracted retrospectively from 9 predefined spatial locations within the water cube for comparison with prior MC study results and TG‐43‐based Oncentra data on the absolute dose level at the same air‐kerma strength and dwell time with difference in %: $100\times \left(D_{\mathrm{Reference}}-D_{MC}\right)/D_{MC}$ ^16^, ^20^, ^22^.

#### 2.4.2 Commissioning level 2

Level 2 validation was performed using an interstitial HDR breast test case, a computational patient phantom model derived from an actual accelerated partial breast irradiation (APBI) treatment^23^. The test case involved 10 flexible plastic catheters and 79 dwell positions of a generic ^192^Ir source. CT images (512 × 512 × 40 with voxel resolution 0.0656, 0.0656, 0.3000 cm) and RT plan were provided by the joint AAPM/IROC Houston Registry of Brachytherapy Sources^24^. MC simulations were carried out with TL dose scoring throughout a dose scoring volume of 25 × 25 × 9 cm^3^ using the *egs_brachy* built‐in Flexisource ^192^Ir source model and spectra of ‘Ir192_NNDC_2.6_line’, rather than the generic source model. Calculations utilized 1×10^9^ histories and 1 mm isotropic voxel spacing. Benchmark comparisons on the absolute dose level were performed against the Registry with MATLAB, where RTSTRUCT and CT images were resampled and aligned with RTDOSE with a dose grid of isotropic 1 mm. Spatial dose agreement was assessed via three‐dimensional gamma index analysis using criteria of 1 mm/1% with 10%, 25% and 100% threshold relative to the prescription dose.

### 2.5 Data analysis

For each patient, RT plan and corresponding CT images were exported from the TPS and imported into the BrachyPlanCheck software tool to automatically generate input files for MC simulations. A set of standardized DVH parameters was employed, which are recommended by the American Brachytherapy Society^25^. The anatomical structures evaluated included CTV, while bone, skin and testes were designated as OARs. Skin was delineated by applying a 3 mm contraction to the external patient contour to approximate superficial tissue exposure. Data analysis was conducted using MATLAB, where RTSTRUCT and CT images are resampled and aligned with RTDOSE with a dose grid of isotropic 1 mm. Histograms and axial, coronal, and sagittal views of dose difference maps and gamma maps were generated. Valid voxels within the patient contour were analyzed individually to determine uncertainty for each contoured structure.

## 3 RESULT

### 3.1 Commissioning level 1: Point dose comparison in homogeneous water phantom

Table 3 summarizes the absolute doses at the 9 predefined locations with dwell time of 44.7 s and Air‐Kerma strength of 28744.48 cGy cm^2^ / h, as well as percentage differences between the TL dose scoring method and other datasets. TL dose scoring method with 1 × 10^9^ histories yielded point dose differences ranging from −0.4% to 0.9% with statistical uncertainty of 0.55%, with 7 points showing deviations within ± 0.5% relative to High Energy Brachytherapy Source Dosimetry Working Group (HEBDWG) reference values^22^. The TL dose scoring method also showed consistency across all other reference datasets. Relative to the *Geant4* study^16^, the dose differences span from –2.3% to 0%, with the smallest deviation at point A2 (5,0) and the largest negative deviation at point A7 (0,15). Comparison with the *EGSnrc* study^20^, it shows differences ranging from –0.5% to +1.7%, with the highest deviation at point A2 (5,0). When compared to the TG‐43‐based Oncentra data, the differences range from –1.9% to +0.5%, with the largest discrepancy at point A8 (0,–20).

**TABLE 3:** Validation of BrachyPlanCheck (x‐axis = radial, z‐axis = longitudinal).

	(x,z)	BrachyPlanCheck	GEANT4^16^	EGSnrc^20^	HEBDWG^22^	Oncentra TG‐43
Point	*in mm*	(cGy)	Diff(%)	Diff(%)	Diff(%)	Diff(%)
A1	(10,0)	396.4	−0.1	0.0	0.2	0.2
A2	(5,0)	1534.7	0.0	1.7	0.2	−0.2
A3	(−10,10)	194.0	−0.3	−0.1	−0.2	0.5
A4	(15,−10)	121.6	−0.2	0.0	−0.1	−1.0
A5	(−20,0)	100.8	−0.5	−0.5	−0.4	−0.8
A6	(−25,−20)	38.6	−0.8	−0.1	−0.1	−1.4
A7	(0,15)	111.5	−2.3	0.6	0.9	−1.5
A8	(0,−20)	52.3	−0.9	−0.2	−0.4	−1.9
A9	(25,15)	46.8	−0.4	0.8	0.7	−0.2

### 3.2 Level 2 commissioning: Dose distribution comparison in heterogeneous breast phantom

**FIGURE 1:**
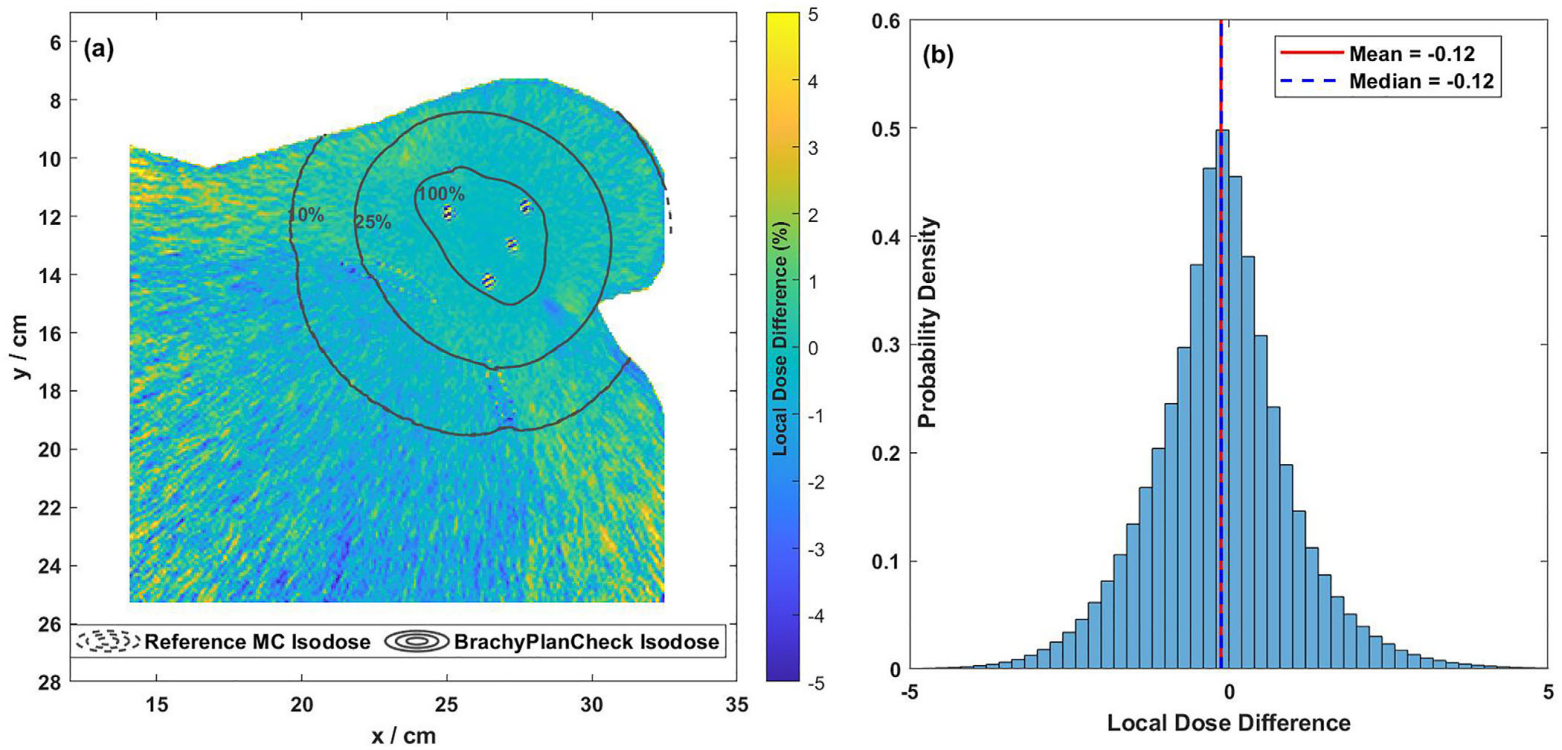
Validation of BrachyPlanCheck with Brachytherapy Source Registry HDR Breast Test Case: (a) Axial views of the local dose difference maps between reference MC dose and MC simulations using BrachyPlanCheck. (b) Histogram of local dose differences was generated across all voxels within the external contour that received non‐zero dose values, without applying any threshold. The colorbar represents the percentage difference relative to the reference MC dose: $100\times \left(D_{\mathrm{MC}}-D_{\mathrm{Reference}-\mathrm{MC}}\right)/D_{\mathrm{Reference}-\mathrm{MC}}$. Isodose lines for 10%, 25%, and 85% of the prescription dose are shown for both reference MC (dashed gray) and MC simulation using BrachyPlanCheck (solid black).

In BrachyPlanCheck commissioning level 2, gamma index analysis was conducted in three dimensions. MC simulations using BrachyPlanCheck with TL dose scoring method yielded high gamma passing rates of 99.9% for criteria of 1 mm/1% with statistical uncertainty of 0.45% and threshold of 10% prescription dose. Excellent gamma agreement is observed throughout the dose scoring volume. Voxels that failed the 1%/1 mm gamma criterion are clustered near the source positions, where dose gradients are steep. Figure 1a presents a local dose difference map comparing MC simulation using BrachyPlanCheck with Registry MC reference data, and Figure 1b presents a histogram of local dose differences across all voxels within the external contour that received non‐zero dose values, without applying any threshold. Both the mean and median values are –0.12%, with a standard deviation of 1.12%.

Within the 100% isodose line, the gamma passing rate (1%/1 mm criteria) was 97.7%, with a local dose difference of –0.20%  ±  1.75%. The 95% confidence interval (CI) for the mean difference was [–0.213%, –0.182%] while the CI for the standard deviation was [1.742%, 1.764%]. The mean statistical uncertainty was 0.19%. Within the 25% isodose line, the gamma passing rate increased to 99.6%, with a local dose difference of –0.06%  ±  0.84%. The 95% CI for the mean was [–0.062%, –0.056%], and the CI for the standard deviation was [0.835%, 0.839%]. The mean statistical uncertainty was 0.31%. Within the 10% isodose line, the gamma passing rate further increased to 99.9%, with a local dose difference of –0.05%  ±  0.78%. The 95% CI for the mean was [–0.052%, –0.049%], and the CI for the standard deviation was [0.781%, 0.784%]. The mean statistical uncertainty was 0.45%.

### 3.3 Comparison of dose distributions using Oncentra TG‐43 and MC simulations using BrachyPlanCheck

**FIGURE 2:**
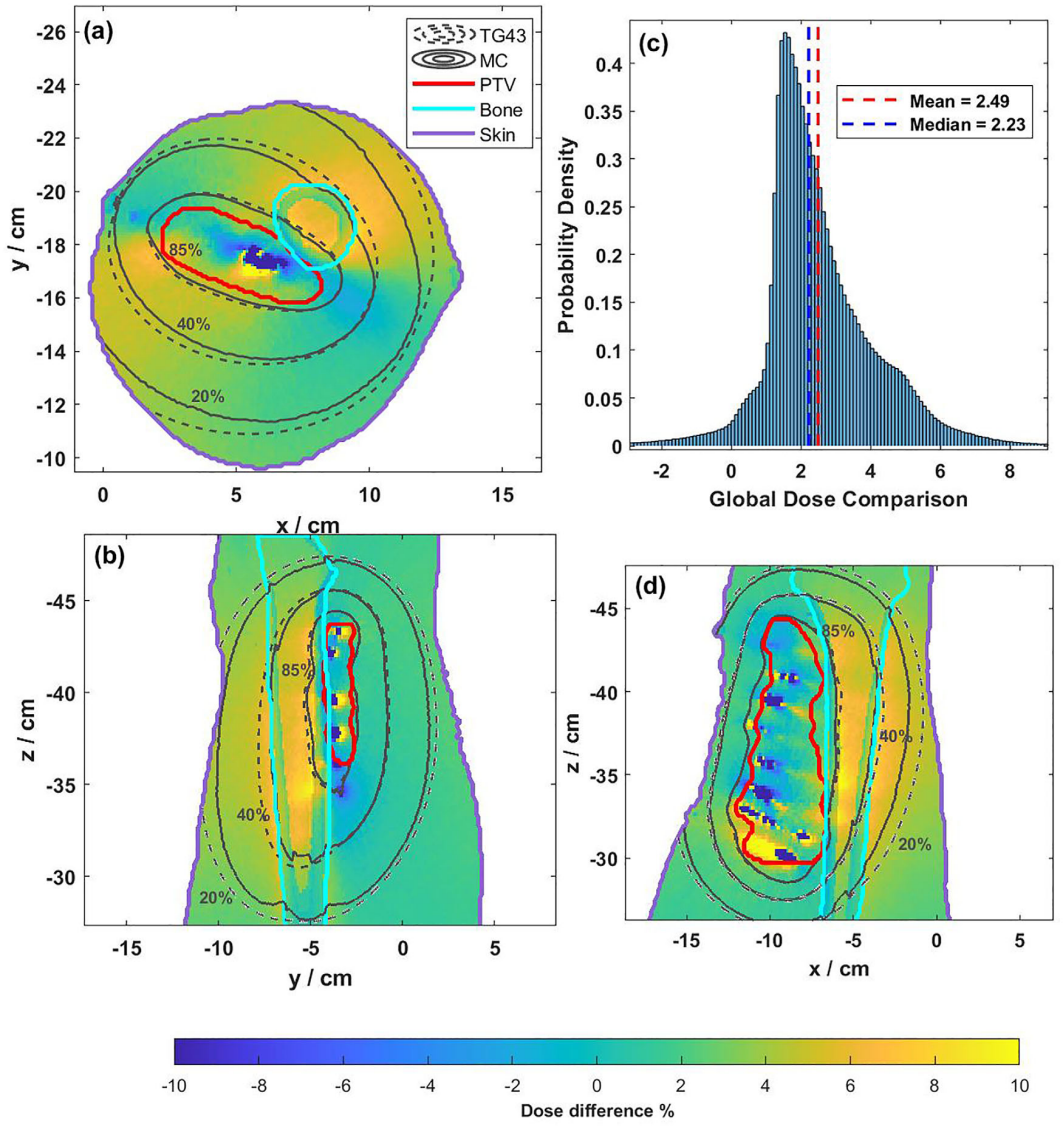
Global dose difference distribution between Oncentra TG‐43 and MC simulations using BrachyPlanCheck for the representative patient 2. (a) Axial,(b) sagittal, and (d) coronal views of the dose difference maps. (c) Histogram of global dose differences was generated across all voxels within the external contour that received non‐zero dose values, without applying any threshold. The colorbar represents the percentage difference relative to the prescription dose: $100\times \left(D_{\mathrm{TG}43}-D_{\mathrm{MC}}\right)/D_{\mathrm{prescription}}$. Isodose lines for 20%, 40%, and 85% of the prescription dose are shown for both Oncentra TG‐43 (dashed gray) and MC simulations using BrachyPlanCheck (solid black).

**FIGURE 3:**
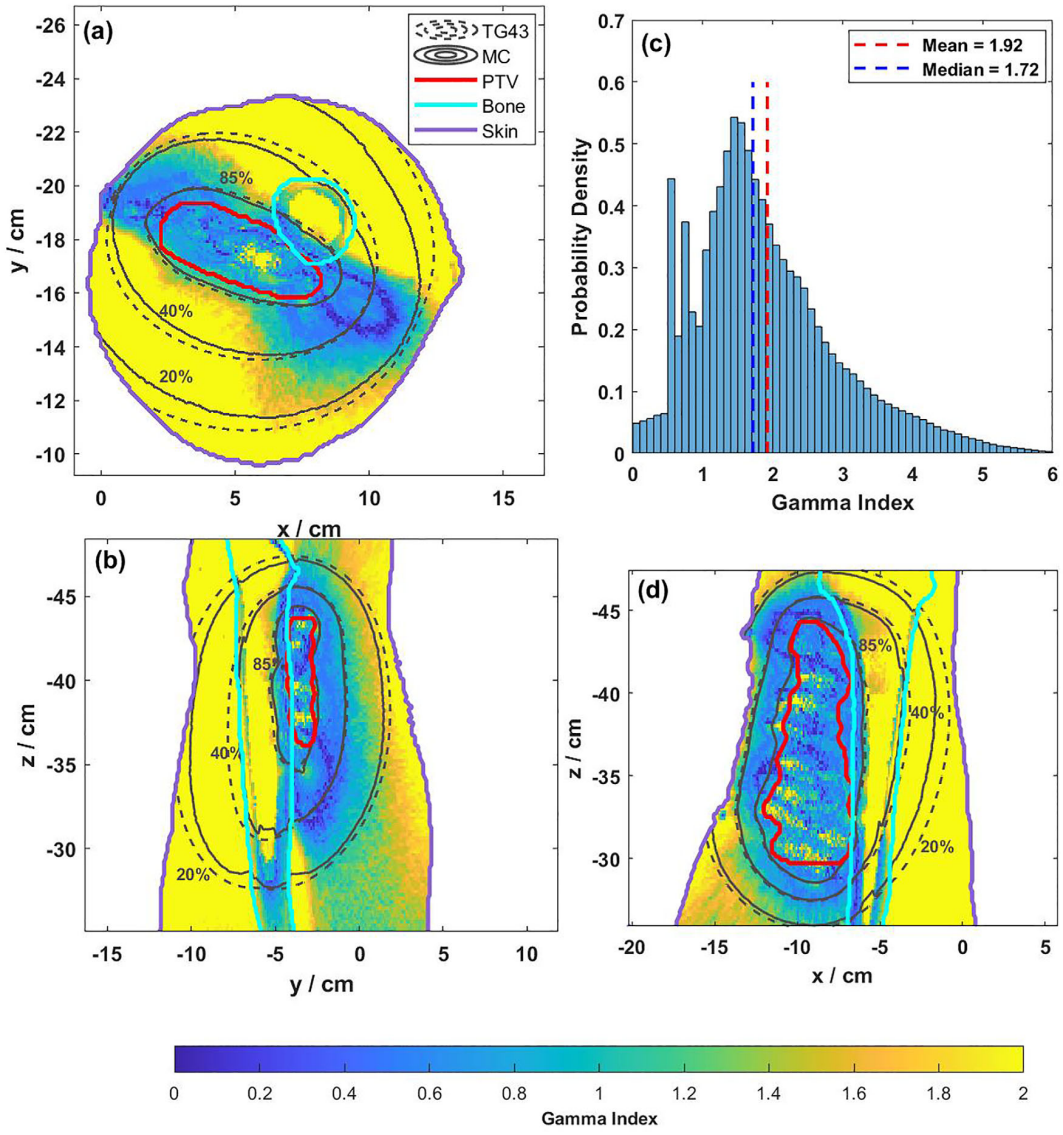
Gamma maps calculated with Oncentra TG‐43 and MC simulations using BrachyPlanCheck for the representative patient 2, where the dose percentage difference for gamma was calculated with a reference value of 100% prescription dose and with a threshold of 10% prescription dose. (a) Axial,(b) sagittal, and (d) coronal views of the dose difference maps. (c) Histogram of gamma index values. The colorbar represents the gamma index, with values below 1 indicating agreement within the specified criteria of 1%/1 mm. Isodose lines for 20%, 40%, and 85% of the prescription dose are shown for both Oncentra TG‐43 (dashed gray) and MC simulations using BrachyPlanCheck (solid black).

A representative patient case, patient 2, with a prescribed dose of 47.25 Gy, is chosen to show a comparison of Oncentra TG‐43 and BrachyPlanCheck dose calculation. The maps of global dose difference between Oncentra and MC simulations using BrachyPlanCheck are shown in Figure 2. The analysis yields a mean global dose difference of 2.49%, indicating a slight overestimation by Oncentra. Figure 3 presents the gamma maps comparing Oncentra TG‐43 and BrachyPlanCheck results, using a 1%/1 mm criterion with a threshold 10% prescription dose, and the reference dose for gamma calculation is 100% of the prescription. Spatial analysis reveals that the discrepancy is most pronounced with the area further from the source. Isodose lines at 85%, 40%, and 20% of the prescription dose further emphasize these differences.

**FIGURE 4:**
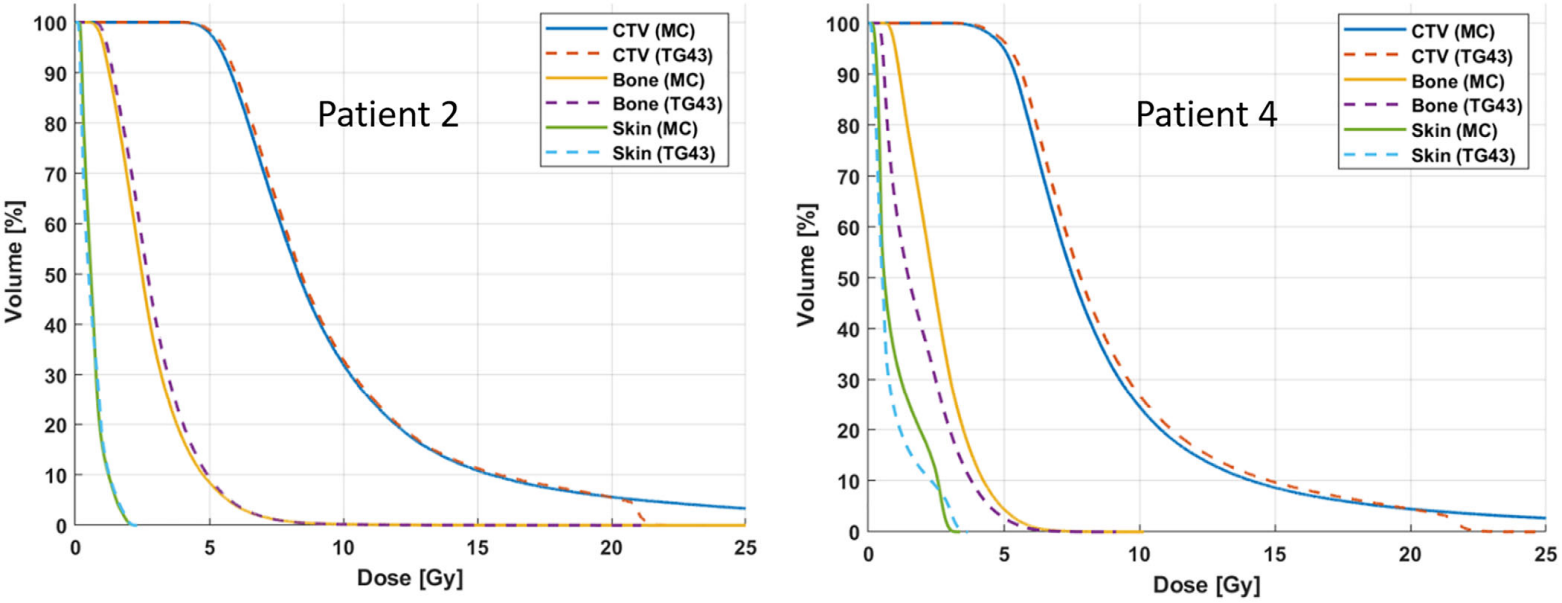
DVH comparison of Oncentra TG‐43 and MC simulation using BrachyPlanCheck for 2 lower extremity malignancy patients. Left: Patient 2. Right: Patient 4.

In the extremity malignancies cohort, MC simulations using BrachyPlanCheck consistently yield lower dose estimates for CTV compared to TG‐43‐based calculations, as shown in Table 4. The mean value of percentage differences in DVH parameters are 3.74% for D90, 3.90% for D95, 2.60% for V100, and 6.33% for V150. The largest deviation of CTV metrics is observed in Patient 7, with a 10.50% difference in V150. For bone, dose differences range from −1.95% to 2.63% for D1cc and from −0.69% to 3.03% for D0.1cc, with arithmetic means of 0.82% and 1.19%, respectively. Skin shows larger dose differences, with D2cc ranging from 1.56% to 12.67% and D0.1cc ranging from 0.91% to 11.67%. The most pronounced discrepancy is observed in Patient 4, who exhibits increases of 12.67% and 11.67% in D2cc and D0.1cc, respectively. Additionally, Patient 8 presents a notable outlier in testes dose, with a 13.70% difference. Figure 4 depicts the TG‐43 and MC dose calculation DVH of the CTV, skin, and bone for two representative cases.

**TABLE 4:** Dosimetric differences between Oncentra TG‐43 and BrachyPlanCheck for the extremity malignancies patient cohort.

Difference in % TG‐43 and BrachyPlanCheck			CTV				Bone		Skin		Testes
No.pt	fx	Dose (Gy)	D90	D95	V_100_	V_150_	D_1cc_	D_0.1cc_	D_2cc_	D_0.1cc_	D_2cc_
1	10	54.0	2.75	2.64	1.75	4.27	−0.67	0.00	6.20	5.35	
2	9	47.3	2.41	2.60	1.00	3.07	1.59	1.34	3.03	0.91	
3	10	42.0	3.59	3.58	0.96	7.14	2.63	3.03	9.73	8.50	
4	10	54.0	4.98	5.41	3.82	10.36	2.34	2.98	12.67	11.67	
5	10	54.0	1.48	1.65	1.11	2.93	1.81	0.61	1.56	3.56	
6	10	54.0	3.14	3.36	2.00	4.68	−1.95	−0.69	8.99	6.96	
7	9	51.3	4.31	4.63	1.17	10.50	−1.10	−0.51	7.93	5.88	
8	10	55.0	7.25	7.37	9.01	7.71	1.92	2.74	7.26	4.01	13.70
maximum			7.25	7.37	9.01	10.50	2.63	3.03	12.67	11.67	
minimum			1.48	1.65	0.96	2.93	−1.95	−0.69	1.56	0.91	
Arithmetic mean			3.74	3.90	2.60	6.33	0.82	1.19	7.17	5.86	
			Note: Difference in %: $100\times \left(\frac{D_{\mathrm{TG}43}}{D_{\mathrm{MC}}}-1\right)$								

### 3.4 Metallic Radiomarkers Override

Table 5 presents DVH statistics for CTV, bone, and skin in the lower limb cancer patient cohort, comparing TG‐43 and MC–based dose calculations with or without overriding CT number for metallic radiomarkers. Mean value with standard deviation is reported for CTV metrics (D90, D95, V100, V150), bone high dose (D1cc, D0.1cc), and skin high dose (D2cc, D0.1cc). Percentage difference of TG‐43 and MC_override,_ relative to MC_original_, with the Wilcoxon signed‐rank test p‐value, is also listed below, where TG‐43 consistently yields higher dose estimates. The largest deviations are observed in skin (D2cc, +7.30%, *p* < 0.01) and CTV (V150, +6.24%, *p* < 0.01), indicating a systematic overestimation of TG‐43 compared with MC simulation. MC_override_ shows differences generally below 0.7% across CTV, bone, and skin DVH metrics compared with MC_original_.

Table 6 reports the dosimetric differences between MC simulations with and without CT number override for metallic radiomarkers in the extremity malignancy patient cohort, for each individual. Differences are expressed as percentages relative to the MC_original_. All DVH metric differences are less than 2.5% except Patient 5 skin D_2cc_ of −6.64% and D_0.1cc_ of −3.20%.

**TABLE 5:** DVH statistics for the extremity malignancies patient cohort.

Dataset	CTV				Bone		Skin	
	D90 (Gy)	D95 (Gy)	V100 (%)	V150 (%)	D1cc (Gy)	D0.1cc (Gy)	D2cc (Gy)	D0.1cc (Gy)
MC_original_	5.38 ± 0.74	4.95 ± 0.74	89.66 ± 12.28	46.17 ± 10.60	5.62 ± 1.45	7.32 ± 2.83	2.98 ± 0.93	3.57 ± 1.59
TG‐43	5.58 ± 0.74	5.13 ± 0.74	91.71 ± 10.87	49.05 ± 11.12	5.69 ± 1.53	7.42 ± 2.90	3.20 ± 1.01	3.77 ± 1.64
Diff %	3.62%	3.79%	2.29%	6.24%	1.09%	1.38%	7.30%	5.68%
p value	0.008	0.008	0.008	0.008	0.109	0.078	0.008	0.008
MC_override_	5.38 ± 0.74	4.94 ± 0.74	89.69 ± 12.18	46.34 ± 10.69	5.64 ± 1.48	7.34 ± 2.85	2.96 ± 0.95	3.55 ± 1.60
Diff %	0.02%	−0.05%	0.03%	0.38%	0.36%	0.26%	−0.67%	−0.42%
p value	0.281	0.750	0.180	0.109	0.562	0.062	1.000	1.000
	*Note: Difference in % relative to* MC_original_ *with the Wilcoxon signed‐rank test p‐value*							

**TABLE 6:** Dosimetric differences between with and without override for the extremity malignancies patient cohort.

Difference in % MC_override_ and MC_original_	CTV				Bone		Skin		Testes
Patient index	D90	D95	V_100_	V_150_	D_1cc_	D_0.1cc_	D_2cc_	D_0.1cc_	D_2cc_
1	0.18	0.20	0.07	0.24	−0.45	−0.37	0.00	−0.23	
2	0.17	0.19	0.08	0.44	0.24	0.37	0.00	0.00	
3	0.42	0.22	0.06	0.78	0.38	0.41	0.00	0.00	
4	0.37	0.40	0.38	1.18	0.47	0.52	0.33	0.32	
5	−0.92	−1.24	−1.40	1.15	2.41	0.73	−6.64	−3.20	
6	0.35	0.37	0.26	0.89	−1.73	−1.04	0.00	0.00	
7	0.15	0.17	0.07	0.94	0.00	0.26	0.30	0.51	
8	0.75	0.57	0.79	0.87	0.17	0.14	0.64	0.43	0.68
maximum	0.75	0.57	0.79	1.18	2.41	0.73	0.64	0.51	
minimum	−0.92	−1.24	−1.40	0.24	−1.73	−1.04	−6.64	−3.20	
Arithmetic mean	0.18	0.11	0.04	0.81	0.19	0.13	−0.67	−0.27	
	Note: Difference in %: $100\times (\frac{D_{\mathrm{MC}\;\mathrm{override}}}{D_{\mathrm{MC}}\;\mathrm{original}}-1)$								

## 4 DISCUSSION

This study demonstrates the fidelity of the in‐house MC‐based dose verification tool BrachyPlanCheck, built upon the *egs_brachy*. By involving CT‐based material mapping via CT number thresholds, the tool enables dose calculation with tissue heterogeneity. The commissioning results confirm excellent agreement with the reference point dose from HEBDWG in level 1 and the reference dose distributions from the AAPM/IROC Houston Registry in level 2. This work evaluates MC dose verification for extremity malignancies brachytherapy treatment, a site rarely investigated in previous MC‐based studies.

Although BrachyPlanCheck shares many similar core features with *eb_gui*, which was developed in C++, it introduces differences in phantom configuration. Specifically, BrachyPlanCheck enables the use of smaller, high‐resolution dose scoring volumes, which means fewer voxels for dose scoring, reducing the use of RAM for a batch run, allowing more batch runs simultaneously, and reducing computational time. eb_gui has metal artifact reduction (MAR) with simple threshold replacement, which is intended to be used to override high‐density materials enveloping the source. BrachyPlanCheck shares the same function and features as the localization of metallic radiomarker, with an expansion of 3 mm from the centroid to account for the partial‐volume and blooming effects. BrachyPlanCheck is developed with MATLAB, making it a practical alternative for researchers and clinicians for *eb_gui* and cross‐checking.

In commissioning level 1, the most significant deviation occurred with *Geant4*,^16^ showing a –2.3% difference at point A7 (0,15), a central high‐dose region. In addition, *Geant4* consistently reports lower dose values than BrachyPlanCheck across all evaluated points. This systematic bias suggests a potential discrepancy between *Geant4* and *egs_brachy*, which might be attributed to differences in physics modeling and dose scoring. In contrast, *EGSnrc* ^20^ showed a maximum difference of +1.7% at point A2 (5,0), a high‐dose zone where statistical noise is low. However, the difference at this point between BrachyPlanCheck and the other datasets is minimal. The TG‐43‐based Oncentra values exhibited the widest discrepancy of –1.9% at point A8 (0,–20), which is also located in a steep gradient region. The commissioning level 1 data in this study shows the best agreement with HEBDWG reference values,^22^ which combined data from *EGSnrc* ^20^ and *Geant4*.^16^

In BrachyPlanCheck commissioning level 2, the voxels with larger differences shown in Figure 1, located near the source, can be partly attributed to the steep dose fall‐offs relative to the 1 mm dose resolution. It should also be noted that BrachyPlanCheck employs the detailed Flexisource ^1^ ^9^ ^2^Ir model, whereas the Registry reference dataset is based on a generic ^1^ ^9^ ^2^Ir source model.

Across several studies, systematic overestimation of the TG‐43 dose calculation algorithm is observed in terms of multiple DVH metrics compared to MC simulation. A study utilized the ALGEBRA MC platform, which evaluated prostate, chest wall, and breast brachytherapy, where TG‐43 showed the most significant increase in chest wall cases CTV with V100 (up to 13.1%), V150 (up to 26.8%), and a modest increase in D90 and D50 (up to 7.4% and 6.1%).^26^ In another study focusing on patient‐level comparison of 10 breast brachytherapy cases with *EGSnrc* MC code, TG‐43 on average overestimated CTV V150 by 20.5% (from 12.2 to 24.4%), skin D1cc by 6.8%, rib D0.5cc by 3.5%, and heart D1cc by 2.6%.^27^ In our study, which focused on extremity malignancy cases, comparison between Oncentra TG‐43 and MC simulations using BrachyPlanCheck demonstrated similar trends shown in Table 4: Oncentra TG‐43 overestimated CTV metrics such as D90 and D95 by up to 7.25% and 7.37%, V100 and V150 by up to 9.01% and 10.50%. For the organs at risk, TG‐43 dose calculation showed elevated skin dose (up to 11.7% in D0.1cc and 12.7% in D2cc) but minimal differences for bone (< 3.10% in D0.1cc and D1cc), which is expected since skin high‐dose regions lie near air that TG‐43 models as water leading to additional scatter and overestimation, whereas bone high‐dose regions are deeper within the body with less pronounced such effect. Specifically, the patient8's testes D2cc was overestimated by up to 13.7%, and due to the dose constraint in testes D2cc during treatment planning, CTV V100 was limited to 65.8%, and the CTV D90 reached only 78% of the prescription dose. If a secondary dose verification tool such as BrachyPlanCheck were applied in this case, it would justify scaling up dwell times to improve target coverage while fulfilling the testes D2cc dose constraint. Additionaly, in our study, differences in dose‐reporting medium, dose‐to‐water ($D_{w}$) in TG‐43 versus dose‐to‐medium in medium ($D_{m,m}$) in MC, also contribute to the observed discrepancies, particularly in high‐density tissues such as bone. To conclude, the overestimation of TG‐43 dose calculation across studies and anatomical sites highlights its limitation, particularly for significant tissue heterogeneity.

The previous study focusing on cervical cancer cases with TG‐43 significantly overestimating rectal D0.1 cm^3^ by up to 32%, D1.0 cm^3^ by 29%, and D2.0 cm^3^ by 28% compared with *EGSnrc* MC simulations results also shows that bladder dose differences were minimal.^5^ This is reasonable, as the presence of air in those cases introduces significant heterogeneity, primarily affecting the dose to the rectum rather than the bladder due to its location. The large differences observed are also reasonable, as DVH metrics focusing on hotspot regions involve only small‐volume voxels. However, another study stated that the TPS Oncentra CTV D90 value of a nasopharyngeal cancer brachytherapy treatment case is 40% higher than the MC simulations with *EGSnrc*,^4^ which significantly differs from the results of all the studies mentioned above. While the study observed good agreement between *EGSnrc* and TG‐43 in a homogeneous water phantom, it did not perform benchmarking in a heterogeneous medium before applying the MC simulations to clinical scenarios. This direct transition from water to anatomic geometries with heterogeneity may have introduced inaccuracies—potentially due to incorrect material settings or data analysis —which could explain the unusually high dose discrepancies reported.

In Figure 2 and Figure 3, TG‐43 isodose line contours consistently extend beyond those produced by MC simulations using BrachyPlanCheck, particularly in the region at greater distances from the source. Similar findings have been reported in other studies, in which TG‐43 contours overestimate dose coverage compared with advanced algorithms. However, some study results show the MC simulations isodose line contour extends beyond TG‐43 in the region of the lung.^13^, ^28^ A practical approach to evaluating the necessity of advanced algorithm implementation may involve assessing the region of interest based on its distance from the source and the heterogeneity of the surrounding tissue.

The overall dosimetric impact of metallic marker override was clinically insignificant for most patients, as the comparison results in Table 6 confirm that radiomarker overrides have a limited impact on MC dose calculation. However, adjustment should be considered when artifact geometry overlaps with dose‐sensitive regions like skin D2cc in this study, as one selected case revealed the largest discrepancy in skin dose observed in Patient 5, where D2cc shows a −6.64% difference. For patient 5, several metallic radiomarkers were located near the skin high‐dose region, and overriding them reduced the skin D2cc by eliminating additional scatter. This suggests that localized high‐density materials may lead to dose inaccuracy if left uncorrected, warranting override. On the contrary, similar results were observed in another study where low‐density materials, specifically air pockets, were considered.^29^ The most prominent deviation in CTV dose metrics was also found in Patient 5, where significantly more radiomarkers were encompassed within the target contour. Their aggregated contribution led to a noticeable shift in DVH metrics, suggesting that extensive inclusion of metallic radiomarkers may influence estimates of total target dose. To conclude, if radiomarkers are significantly clustered near critical structures, especially when the OAR hot spot region is concerned, CT number override to more physiologically realistic values enhances dosimetric accuracy in MC simulations.

AAPM TG‐138 recommends evaluating both statistical (Type A) and systematic (Type B) components.^30^ TG‐186 further highlights a range of dosimetric uncertainty sources in model‐based dose calculation algorithms (MBDCA), including source and applicator modeling, grid resolution, patient geometry, and CT‐to‐density conversion, with responsibilities divided between vendors and physicists.^3^ In our study, Type A uncertainty was 0.45% for voxels with dose larger than 10% of the prescription dose in commissioning level 2. For patient cases, mean statistical uncertainties were up to 0.7% for CTV, 1% for bone, and 2% for skin. Type B uncertainties were not individually quantified but are estimated at ∼3% based on published reports.^18^, ^31^ Combining Type A (∼0.5%) and Type B (∼3%) with a coverage factor of k=1 yields an overall uncertainty of ∼3%, supporting the reliability of our MC dose calculation.

This study has several limitations. First, the cohort size is relatively small, consisting of only 8 patients, which may limit the generalizability of the findings. Second, the use of the tracklength method without charged‐particle transport introduces uncertainty in dose calculation near material interfaces, where significant heterogeneity is present, and the collision kerma approximation is applied to reduce computation time. Third, streaking artifacts from metallic radiomarkers were not specifically addressed, as their impact was considered less critical than the unwanted presence of metallic radiomarkers in phantom modeling for dose calculation, although TG‐186 recommends handling such artifacts to improve accuracy, and this remains a potential source of error.^3^

## 5 CONCLUSION

This study presents a dose verification tool, BrachyPlanCheck, for brachytherapy with *egs_brachy* and evaluates it on extremity malignancies ^192^Ir HDR interstitial brachytherapy cases compared with Oncentra TG‐43. Validated by phantom and patient studies, BrachyPlanCheck has demonstrated robust and extensible performance for independent dose verification, with applications ranging from algorithm commissioning to retrospective analysis. BrachyPlanCheck's voxel‐based modeling and localized high‐resolution subvolumes enabled more accurate dose evaluation. For lower limb cancer ^192^Ir HDR interstitial brachytherapy treatment plans, the retrospective study shows that TG‐43 significantly overestimates OAR dose and CTV coverage, underscoring the need for caution when relying on TG‐43 for prescription. These findings may inform adjustments in treatment prescription and plan optimization strategies, though any changes require careful review of historical clinical data and recognition that dosimetric uncertainties must be considered. Clinical integration also demands attention to computational requirements, workflow adaptation, and staff training. Future developments may include extension to broader treatment sites, refinement of material assignment, and incorporation of uncertainty quantification to further strengthen dosimetric confidence.

## AUTHOR CONTRIBUTIONS

Bin Yang (Corresponding Author): Led the project, provided resources and conceptual guidance, supervised the work, and contributed to manuscript review and final approval. Peixiong Li (First Author): Developed the software, performed commissioning, conducted Monte Carlo dose calculations, carried out data analysis, drafted the manuscript. Chi Wah Kong, Rihui Zhang, Dejun Zhou, Chenyu Huang, Tin Lok Chiu, Pak Hang Nam, Chi To Yung, Tien Yee Chang, Siu Ki Yu: Provided technical support, participated in discussions, and contributed to manuscript refinement.

## FUNDING INFORMATION

This research did not receive any specific grant from funding agencies in the public, commercial, or not‐for‐profit sectors.

## CONFLICT OF INTEREST STATEMENT

The authors declare no competing interests.

## ETHICAL APPROVAL

This study was conducted in accordance with ethical standards and approved by the Hong Kong Sanatorium & Hospital Research Committee (Approval No. RC‐2025‐02).

## Data Availability

Due to patient confidentiality, the datasets generated and/or analyzed during this study are not publicly available. Data supporting the findings are available from the corresponding author upon reasonable request, subject to institutional approval.
